# What’s new in dry eye disease diagnosis? Current advances and challenges

**DOI:** 10.12688/f1000research.16468.1

**Published:** 2018-12-19

**Authors:** Shruti Aggarwal, Anat Galor

**Affiliations:** 1 Department of Ophthalmology, Bascom Palmer Eye Institute, University of Miami, Miller School of Miami, 900 NW 17th Street, Miami, FL, 33136, USA; 2 Department of Ophthalmology, Miami Veterans Affairs Medical Center, 1201 NW 16th St., Miami, FL, 33125 , USA

**Keywords:** Dry eye disease

## Abstract

Dry eye disease (DED) is a commonly encountered condition in general ophthalmology practice and imparts a significant socioeconomic burden. Despite its prevalence, there remain challenges regarding its diagnosis and management. A major reason behind these challenges is the fact that DED represents an umbrella term that encompasses many different underlying conditions and pathophysiological mechanisms. The purpose of this article is to highlight aspects of DED pathophysiology and focus on targeted diagnostic and therapeutic approaches to this multifactorial, chronic condition.

## Background

With a global prevalence ranging from 20 to 50%, dry eye disease (DED) is a significantly growing health problem worldwide ^1^. DED has been diagnosed in about 16.4 million adults in the US, and 6 million more experience DED symptoms without a formal diagnosis. DED is more prevalent in older individuals and women ^1^. However, it is on the rise among the young as well, and recent studies report DED symptoms in 30 to 65% of office workers ^2, 3^ and 25% of high school students ^4, 5^. DED symptoms in young individuals have been linked to the use of digital devices ^6, 7^ and refractive treatments, including contact lens wear and refractive laser. Also, increased awareness of the association between DED and autoimmune diseases, hormonal changes, and systemic drug therapies has increased the recognition of DED symptoms by physicians in other specialties.

DED has a marked negative impact on the physical and psychosomatic well-being of patients because of discomfort, pain, and altered visual acuity, preventing them from carrying out basic activities of daily living, such as reading, watching television, driving, and working ^1^. Symptom severity correlates positively with patient-reported depression, anxiety, and stress scores ^8– 11^. Increased suicidal ideation in patients with severe DED underscores the extreme impact that DED can have on quality of life ^12^.

DED also exerts a substantial economic impact. One healthcare utility assessment study found that DED symptoms were comparable to those of angina ^13^. The estimated average annual direct cost of ophthalmologist-managed care of one DED patient ranges from $270 USD in France, $530 USD in Japan, and $800 USD in the US to $1100 USD in the UK ^14^. Additionally, the economic burden increases exponentially when loss of work productivity is taken into account. In the US, the annual direct healthcare cost of DED is about $4 billion and loss of productivity accounts for up to $55 billion annually ^1, 15^.

Given the increasing public health importance of DED, there has been significant research in the field of DED and ocular surface health. This article addresses the newly evolving strategies and highlights the persistent gaps in our understanding of this challenging condition and future directions of research.

## Discussion

Our approach to DED has undergone a paradigm shift in the past few decades as new research and the use of sophisticated imaging modalities have improved our understanding of the pathophysiology of DED. The first formal definition of DED, published in 1995 by the National Eye Institute/Industry working group on Clinical Trials in Dry Eye, was “Dry eye is a disorder of the tear film due to tear deficiency or excessive tear evaporation which causes damage to the interpalpebral ocular surface and is associated with symptoms of ocular discomfort” ^16^. One major limitation in this definition is that it suggests that dry eye is one disease. An emerging paradigm in dry eye is the recognition that DED is an umbrella term that represents a spectrum of disease. This led to the new refined definition by a panel of experts in the field at the Tear Film and Ocular Surface Dry Eye Workshop II in 2017: “Dry eye is a multifactorial disease of the ocular surface characterized by a loss of homeostasis of the tear film, and accompanied by ocular symptoms, in which tear film instability and hyperosmolarity, ocular surface inflammation and damage, and neurosensory abnormalities play etiological roles” ^17^.

Inflammation has played a central role in DED given that the disease was first described in individuals with Sjögren’s. Animal models highlighted the central role of inflammation in DED by demonstrating that hyperosmolar stress on the ocular surface triggered the release of inflammatory mediators such as interleukin 1 beta (IL-1β), tumor necrosis factor-alpha (TNF-α), and matrix metalloproteinase 9 (MMP-9) ^18^. Similar inflammatory mediators have been found on the ocular surface of individuals with DED ^19, 20^. The importance of T cells in the pathophysiology of DED was demonstrated by triggering disease in naïve mice solely by the transfer of T cells from affected animals ^21, 22^. However, it needs to be considered that the definition of DED in these mice consisted of corneal epithelial staining, which is a component—but does not represent the full spectrum—of DED in humans. Furthermore, inflammation and resulting ocular surface damage form a vicious cycle in which inflammation both triggers and results from chronic DED.

In humans, however, DED is typically diagnosed by symptoms, an aspect of DED that does not have a good animal model counterpart. Even the symptoms of DED vary; some individuals present with symptoms of pain (described in terms of dryness, burning, aching, tenderness, and foreign body sensation) and others with poor or fluctuating vision. The etiology of these two diverse symptoms suggests two different underlying mechanisms in DED. In the former, nociceptor activation from terminal nerve endings on the cornea and conjunctiva leads to symptoms of pain; in the latter, an unhealthy tear film and ocular surface disruption result in fluctuating or poor vision. Adding to the complexity, it is well known that symptoms of DED do not correlate well with observed ocular surface signs ^23^. Given this reality, to understand DED in the context of an individual patient, the treating physician needs to properly diagnose and treat the specific pathophysiologic mechanisms that drive symptoms in a particular patient.

## Tear film stability

The tear film comprises three layers: an innermost mucin layer composed of gel and soluble mucins secreted by conjunctival goblet cells that adhere the tear film to epithelial cells, a middle aqueous layer secreted by lacrimal and accessory glands to provide hydration and lubrication, and an outer lipid layer secreted by Meibomian glands to decrease tear evaporation ^24, 25^. Recently, a two-tiered model in which the mucin and aqueous interact in a muco-aqueous layer was proposed ^26^. Tear production is controlled by sympathetic and parasympathetic stimulation of the lacrimal glands, which in turn is controlled by a neural reflex arc originating from the ocular surface ^27, 28^. Innervation of the Meibomian glands and goblet cells is less well understood but is also likely under parasympathetic control ^29^.

## Aspects to be considered in patients with dry eye disease symptoms

### Inflammation

Given the central role of inflammation in DED, it is important to look for an underlying inflammatory component. This is most common in individuals with systemic immune problems—such as in Sjögren’s and graft-versus-host disease—in which DED is one manifestation. InflammaDry (Quidel, San Diego, CA, USA) is a point-of-care test that qualitatively measures levels of ocular surface MMP-9 ^19^. The intensity of the pink stripe gives an idea of the amount of MMP-9 on the ocular surface. Researchers have also used confocal microscopy to detect dendritic cells in the cornea, but the test is not often used in clinical practice ^30^. Oftentimes, this presentation is accompanied by lacrimal gland dysfunction and low tear production. Other rare causes of low tear production include damage to the lacrimal glands by trauma or radiation. Several agents—including topical corticosteroids, cyclosporine (Restasis, Allergan, Dublin, Ireland), and lifitegrast (Xiidra, Shire, Lexington, MA, USA)—are used to target inflammation in DED.

### Meibomian gland health

Meibomian gland disease (MGD) is recognized as a major contributor to DED ^31^. Meibomian glands secrete lipids containing non-polar lipids, polar lipids, and phospholipids which increase the viscosity of the tear film and prevent evaporation. Two major theories underlie the development of MGD. In one, chronic inflammation in response to bacterial colonization of eyelids, dust, and other environmental factors leads to hyper-keratinization of terminal ducts and subsequent glandular obstruction ^32, 33^. Another theory involves altered expression of the nuclear receptor, peroxisome proliferator-activated receptor gamma (PPARγ). PPARγ is a nuclear receptor protein involved in regulating meibocyte differentiation and lipid biosynthesis, contributing to the formation and function of Meibomian glands ^34^. Downregulation of PPARγ is thought to underlie the decreased meibocyte differentiation and lipid synthesis seen in aging and in the setting of other environmental stresses, leading to gland atrophy and a hyposecretory state ^35, 36^. Alteration in levels of systemic sex hormones, glucocorticoids and mineralocorticoids, and other growth factors has also been found to affect meibum quality and viscosity, leading to tear film instability and increased evaporation of tears ^37^. Inflammation can be a secondary part of this process, as fast evaporation can lead to increased tear osmolarity ^27^. Hyperosmolarity leads to apoptosis of epithelial cells, increases oxidative stress, and triggers an inflammatory cascade with upregulation of MMP-9, mitogen-activated protein kinase (MAPK), TNF-α, and various interleukins ^38^. In addition, drugs such as oral retinoic acid have been associated with Meibomian gland loss ^39^.

Meibomian gland dysfunction is typically evaluated in the clinic by evaluating gland inspissation, squeezing the Meibomian glands and rating the quality of extracted meibum, and retro-illuminating the eyelids to look for gland atrophy. The clinical examination can be supplemented by imaging tests—such as LipiView (TearScience, Morrisville, NC, USA) and Keratograph (Oculus, Arlington, WA, USA)—that highlight gland anatomy. In addition, clinicians should determine whether rosacea, including telangiectasias on the skin and eyelids which can associate with MGD, is present. Treatments for MGD include home- and clinic-based lid hygiene treatments and oral and topical antibiotics (for example, doxycycline) which are thought to have an anti-inflammatory effect ^40^.

### Anatomy

Any anatomical disturbances of the eyelid can cause dry eye symptoms, both ocular pain in the setting of nociceptor activation and poor, fluctuating vision due to fast evaporation and corneal epithelial erosions. Common anatomical disturbances of the eye include conjunctival changes (for example, conjunctivochalasis, pterygium, pinguecula, and conjunctival elevation from glaucoma surgery) and eyelid changes (for example, eyelid laxity, ectropion, entropion, and lagophthalmos). These abnormalities should be investigated as part of a dry eye evaluation and corrected as needed.

### Nerve dysfunction

The activation of nociceptors upon exposure to noxious stimuli and cellular injury (from the sources outlined above) can lead to a physiological pain response known as nociceptive pain. A new paradigm in DED is that, in addition to appropriately relaying information about the ocular surface, corneal and conjunctival nerves may become dysfunctional and transmit signals inappropriately, which can also result in sensations of ocular pain (termed neuropathic pain). The clinical correlate behind this idea is that dry eye symptoms can originate from nociceptive sources or neuropathic mechanisms or a combination of the two.

Trigeminal nerve endings may be damaged by various insults, including tear hyperosmolarity, surgical trauma, and air pollution. Chronic injury results in upregulation of voltage-gated ion channels on the terminal nerve endings. This results in abnormal ectopic activity and hyper-responsiveness to stimuli, evoking sensations of dryness, discomfort, and pain. Chronic peripheral nerve stimulation can alter the higher signaling neuronal pathways in a process known as central sensitization. These mechanisms decrease the pain threshold and are responsible for increased perception of pain (hyperalgesia), even to non-painful stimuli (allodynia). This abnormal neuronal excitability can become permanent, and pain can continue even after apparent healing of the damaged tissue ^41^.

We have termed this concept neuropathic ocular pain (NOP) to highlight the fact that pain generation can initiate from nerves on the ocular surface (cornea and conjunctiva) or from secondary and tertiary neurons that connect the ocular surface to the brain. Risk factors for the development of NOP include chronic overlapping pain conditions and migraine, and central sensitization is thought to link the entities ^42, 43^.

There are no gold-standard tests to detect the presence of NOP. We have found certain features to be helpful in suggesting a neuropathic component to ocular pain, including the presence of specific pain descriptors (spontaneous burning pain or evoked pain to wind or light, the ocular equivalents of hyperalgesia and allodynia); risk factors such as chronic widespread pain, migraine, or history of refractive surgery (due to surgical injury in the area); a disconnect between symptoms and ocular signs of disease; and persistent pain after placing a drop of topical anesthesia. Again, confocal microscopy has been helpful in identifying individuals with anatomical abnormalities (via the presence of micro-neuromas) ^44^, but this test is not generally used in routine clinical practice. Treatments for NOP are emerging and include first treating for all nociceptive sources of pain (improving tear and Meibomian gland health). Scleral contact lenses can be used to provide constant lubrication to the ocular surface. In patients with persistent symptoms despite ocular surface optimization, various strategies have been used, borrowing from what is known about the treatment of neuropathic pain outside the eye. This includes oral therapies with α2ϫ ligands (gabapentin and pregabalin), adjuvant stimulation therapies ^45^, injections with botulinum toxin ^46^, and topical therapy with autologous serum tears ^44^. Given the emerging nature of the condition, optimal therapies are still unknown.

## Conclusions

DED is a common, multifactorial condition with a variable clinical presentation and frequent discordance between symptoms and signs of disease. As such, the elucidation of distinct pathways underlying the various clinical phenotypes is critical. Thus, it is essential to perform a standardized examination and to evaluate the different components of DED while considering local findings like MGD and systemic diseases, including autoimmune conditions like Sjögren’s, rosacea, chronic overlapping pain, and migraine. Objective and quantitative diagnostic methods can be used to detect the hallmarks of DED: ocular surface inflammation, epithelial damage, and tear film hyperosmolarity. However, it is important to note that new diagnostic tests are needed to evaluate some aspects of DED, particularly the presence of nerve dysfunction. For optimal management, DED in an individual patient should be subcategorized in terms of underlying pathophysiology and clinical severity and a treatment plan should be tailored with objective tests used to follow therapeutic response.
